# The Mph1 Helicase Can Promote Telomere Uncapping and Premature Senescence in Budding Yeast

**DOI:** 10.1371/journal.pone.0042028

**Published:** 2012-07-27

**Authors:** Sarah Luke-Glaser, Brian Luke

**Affiliations:** Zentrum für Molekulare Biologie der Universität Heidelberg (ZMBH), DKFZ-ZMBH Allianz, Heidelberg, Germany; Tulane University Health Sciences Center, United States of America

## Abstract

Double strand breaks (DSBs) can be repaired via either Non-Homologous End Joining (NHEJ) or Homology directed Repair (HR). Telomeres, which resemble DSBs, are refractory to repair events in order to prevent chromosome end fusions and genomic instability. In some rare instances telomeres engage in Break-Induced Replication (BIR), a type of HR, in order to maintain telomere length in the absence of the enzyme telomerase. Here we have investigated how the yeast helicase, Mph1, affects DNA repair at both DSBs and telomeres. We have found that overexpressed Mph1 strongly inhibits BIR at internal DSBs however allows it to proceed at telomeres. Furthermore, while overexpressed Mph1 potently inhibits NHEJ at telomeres it has no effect on NHEJ at DSBs within the chromosome. At telomeres Mph1 is able to promote telomere uncapping and the accumulation of ssDNA, which results in premature senescence in the absence of telomerase. We propose that Mph1 is able to direct repair towards HR (thereby inhibiting NHEJ) at telomeres by remodeling them into a nuclease-sensitive structure, which promotes the accumulation of a recombinogenic ssDNA intermediate. We thus put forward that Mph1 is a double-edge sword at the telomere, it prevents NHEJ, but promotes senescence in cells with dysfunctional telomeres by increasing the levels of ssDNA.

## Introduction

In the absence of telomerase, telomeres progressively shorten with each cell division and eventually, when they are critically short, get recognized as DNA damage due to the inability to maintain their protective cap structure [1], [2]. Critically short telomeres activate a checkpoint response leading to cell cycle arrest and eventual cellular senescence. In rare instances, cells acquire the ability to maintain their telomeres via a homology-directed repair (HR) mechanism, and thereby evade checkpoint-mediated arrest [3]. In yeast, such cells are referred to as survivors, whereas human cells using HR to maintain telomeres have been named ALT (Alternative Lengthening of Telomeres) cells [4]. Interestingly, although most human cancers up-regulate telomerase, about 15% of human cancers maintain their telomeres through the ALT pathway [5]. BIR has been proposed to be the underlying mechanism in survivor establishment, as yeast mutants unable to perform BIR, *i.e.* lacking the non-essential DNA polymerase ∂ subunit Pol32, are also defective in forming survivors [6]. BIR is specifically initiated at a one-ended break that can arise at a critically short telomere or from a replication fork collapse [7], [8]. The invasion of one end results in the formation of a D (dissociation) loop, whereby a uni-directional replication fork is established and subsequently gets elongated. Indeed, BIR is suppressed at a DSB, where both ends share homology with a template, in order to prevent loss of heterozygosity (LOH) and to allow a more classical gene conversion (GC) reaction to carry out repair of the DSB [7]. Both the yeast homolog of the Bloom helicase, Sgs1, and the exonuclease, Exo1, are able to inhibit the BIR reaction at a double-stranded break in yeast when over-expressed [9].

The budding yeast (*S. cerevisiae*) helicase Mph1 (mutator phenotype 1) is a putative homolog of the Fanconi Anemia protein M (FANCM) [10]. Fanconi Anemia is a heritable disease associated with bone marrow failure, genomic instability and early onset cancer [11]. Fanconi anemia genes are overexpressed in melanoma cells [12] and were implicated in resistance to chemotherapeutics in various tumors [13], [14]. In yeast, deletion, as well as overexpression of *MPH1* leads to genomic instability [15], [16]. Although extensive genetic studies have been carried out with *mph1Δ* mutants [15], [17], the *in vivo* function of Mph1 remains unclear. Interestingly, *in vitro,* Mph1 has the ability to displace D-loops [18] suggesting that it may play a role in either preventing inappropriate recombination events or perhaps in their resolution. Here we have used an overexpression approach to gain insight into Mph1’s *in vivo* function.

## Methods

### Yeast Strains, Plasmids and Culture Media

All yeast strains were grown following standard protocols and using common culture media. The following strains used in these studies are derivatives of BY4741 (*his3Δ1 leu2Δ0 met15Δ0 ura3Δ0*) unless stated otherwise. Double mutants were constructed through standard mating and tetrad dissection procedures, except for the BIR strain, where *MPH1* was deleted by integrating a previously amplified gene cassette. The BIR strain JRL 347 was a gift from the Haber lab. Strains used to assay GC and NHEJ were kind gifts from the Gasser Lab [19]: GC: GA-1080 parental strain from the GAL HO strains, GA-2321 ade3::GALHO MATinc-URA3 and *RAD52* disrupted in GA2321. NHEJ strains: GA-1080 parental strain from the GAL HO strains, JKM179, ku70::URA3 JKM181. WT *MPH1* and *mph1E210Q* were cloned via PstI and SalI into pRS425 (pGal, Leu2 and pGPD, Leu2). A HA-tag was included to check expression by Western Blotting. In order that the C-terminal HA tag does not interfere with Mph1 activity, a flexible RGS (arginine, glycine, serine) linker was introduced immediately after the open reading frame of *MPH1*. The forward primer included the sequence: 5-CGCCTGCAGATGGCTAGTGCAGATGATTAC-3, reverse primer: 5-GCCGTCGACTCACAAAGAAGCGTAATCTGGAACATCATATGGGTAGGCAGAACCTCTAAAATCAGAATCTGAGCCCAG-3. *EXO1* was cloned similarly with a C-terminal HA tag separated from the *EXO1* ORF via an RGS linker into pGPD, Leu2, 2micron. The following primers containing and HindIII or a XhoI were employed: 5′-CGCAAGCTTATGGGTATCCAAGGTCTTCTTCC-3′; 5′- GCCCTCGAG*TCA*CAAAGAAGCGTAATCTGGAACATCATATGGGTAGGCAGAACCTCTTTTACCTTTATAAACAAATTGGG-3′. The plasmids used in the *rad52Δ est1Δ* senescence assay were gifts from the Johnson lab (pAG415GAL-ccdB, PAG415GAL-*MPH1*, centromeric, Leu2).

### Senescence and Survivor Assay in Culture

Heterozygous diploids *est1Δ/EST1* were transformed with plasmids and sporulated in liquid sporulation medium containing 1% potassium acetate and 0.005% zinc acetate. For the liquid growth assay, isogenic single and double mutants were diluted to an OD_600_ of 0.01 and grown in YPD for 24 hours at 30°C. The OD_600_ was determined and the culture was re-diluted to an OD_600_ of 0.01. This procedure was repeated for up to 10 days or until survivors had formed. The number of population doublings was calculated with the following formula: Log2 (OD600/0.01). For the senescence assays in galactose-containing medium population doublings were counted from when plasmid expression by galactose was initiated. For the senescence assay in YPD medium the 25 population doublings on the tetrad dissection plate were taken into account.

### BIR Assay

Cells were grown over night in 1% raffinose medium lacking leucine to maintain the *MPH1* or the empty vector. Saturated cultures were diluted to an OD_600_ = 0.5 and serial tenfold dilutions were spotted on SD-Leu and SGal-Leu plates. After 3 days at 30°C the plates were replica plated onto canavanine and hygromycin plates to confirm that repair occurred via BIR. To quantify the efficiency of BIR, approximately 200 cells of the overnight culture were plated onto SD-Leu and SGal-Leu plates respectively and colonies were counted. Five independent cultures for the vector control and four *GAL-MPH1* transformed colonies were analyzed for their capacity to repair BIR, which was calculated as growth on galactose/growth on glucose.

### HO and NHEJ Assay

Cells were grown over night at room temperature in media lacking leucine and containing 1% raffinose. Cells were diluted to an OD_600_ = 0.5 and tenfold dilutions were spotted on glucose and galactose plates lacking leucine. Cells were grown 2-3 days at 30°C. Deletion of *RAD52* served as a control for a strain defective for repair by homologous recombination (HR), whereas disruption of *KU70* leads to a deficiency in non homologous end joining (NHEJ).

### Telomere PCR

Approximately 10 OD_600_ units of cells were pelleted and DNA was extracted using the puregene yeast kit (Qiagen). 100 ng DNA was denatured for 10 minutes at 96°C and a cytosine (C)-tail was added by terminal transferase (NEB) while incubating for 30 minutes at 37°C. After heat inactivation of the terminal transferase for 10 minutes at 65°C, telomeric DNA was amplified by Phusion polymerase (Finnzymes). The reverse primer annealing with the C-tail was: CGGGATCCG_18_. The following forward primers were used in the PCR reactions: 5-TTAGGGCTATGTAGAAGTGCTG-3 (Y’ of 12L), 5-GCGGTACCAGGGTTAGATTAGGGCTG-3 (1L) and 5-AAATGAGGACTGGGTCATGG-3 (6R). The primers (all 1 µM final) were allowed to anneal for 15 seconds at 63°C followed by 20 seconds of elongation at 68°C (45 cycles). The PCR products were separated on a 1.8% agarose gel. The fragment size was analyzed with the help of Multi Gauge software (Fuji). The primer lengths were substracted and the length distribution was presented graphically.

### ssDNA dot Blot and Southern Blot

For the ssDNA dot blot cultures of cells were grown in raffinose containing medium at 23°C to log phase, 2% galactose was added to induce *MPH1* expression and cells were arrested for three hours with in 20 µg/ml nocodazole (Applichem). Cultures were then shifted to 27°C by adding the appropriate amounts of pre-warmed medium containing nocadozole. DNA was extracted under native conditions (Puregene kit, Qiagen). The 65°C step was omitted to prevent denaturation of DNA. 3 µg of DNA was spotted onto a nylon membrane (Amersham). For the loading control 0.25 µg of DNA was denatured with 0.2 M NaOH for 15′ at 65°C. Membrane was pre-hybridized for 30′ at 47.5°C. The telomeric C- (CACCACACCCACACACCACACCCACA) and G-(GTGGGTGTGGTGTGTGGGTGTGGTG) probes were DIG-labeled (Roche High grade kit) and allowed to hybridize over night at 47.5°C. The membrane was washed twice in 2×SSC 0.1% SDS and twice in 0.5×SSC 0.1%SDS at 47.5°C. The membrane was blocked and hybridized with AP-coupled antibody against DIG (Roche) for 30 minutes at room temperature. The signal was detected by CDP-star solution (Roche) and quantified by the ImageJ software. ssDNA was digested for 2 hours at 37°C by 20 units of Exonuclease I (*E. coli*, NEB) in a total volume of 15 ml.

For Southern blotting the procedure was as described above, but the DNA (25 µg) was denatured for 1 hour at 65°C and digested for 4 h with XhoI prior to loading and separting on a 1.2% agarose gel. The DNA was transferred onto a nylon membrane at 1A for 2 hours at 4°C. Antibody binding, washing and detection were identical to the ssDNA blot.

### Telomere Fusion Assay

Was carried out using Lev1212 and Lev728 (*rap1-(Δ)*) as previously described [20]. Sequences for *HIS4* PCR are:

5-GACGCTCCAGAGGAATCTTA-3 and 5-TTGGTCTGCTCAAAGCCTTC-3. The PCR reaction was carried out by Phusion HF (Finnzymes).

## Results

### Mph1 Abolishes Break-Induced Replication at a DSB, but Allows Survivor Formation

**Figure 1:**
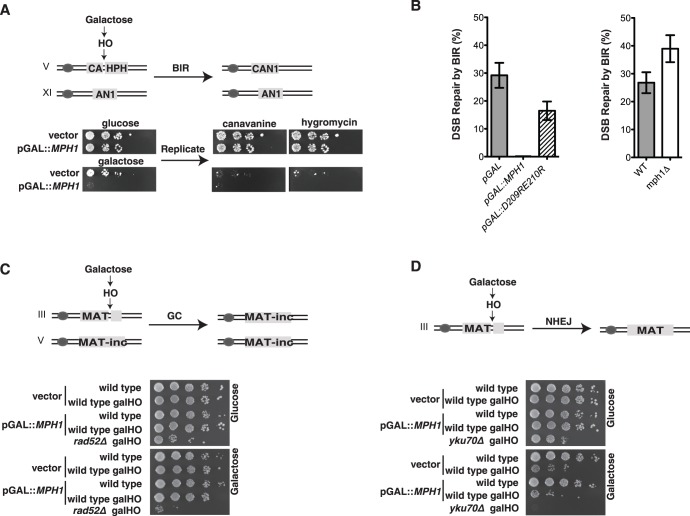
Mph1 inhibits break-induced replication at a DSB. *(A)* Cells harboring the indicated constructs on chromosomes V and XI were grown overnight in raffinose and spotted as 10-fold serial dilutions on either glucose or galactose containing media (to induce an HO DSB). Growth on galactose and subsequent canavanine and hygromycin sensitivity indicates that those cells were able to repair an HO induced DSB via BIR directed repair. This is due to the fact that following BIR the hygromycin resistance cassette (HPH) was lost and the canavanine sensitivity marker (CAN1) was re-constituted due to homology between the “A’s”, (for more detailed description see [9]). *(B)* Cells harboring the constructs described in *(A)* were grown over night in raffinose and spread onto glucose and galactose plates, colonies were counted and the survival ratio of galactose/glucose indicated the percentage of cells that were able to repair a DSB by BIR. Upon overexpression of wild-type *MPH1* cells were unable to repair the DSB via BIR. Mutations in the DEAH domain (D209RE210R) partially restored the ability to perform repair via BIR (left panel). The *mph1Δ* (n = 8) and corresponding WT (n = 4) strains were grown in YPD medium overnight and spread on YPD and YP medium containing 2% galactose. *mph1Δ* cells show an increased rate of repair by BIR right panel. *(C)* An HO cut in the MAT locus results in gene conversion due to the fact that there is homology on both sides of the HO site in the MAT_inc_ locus on chromosome V (upper panel). Cells were grown and spotted as in *(A)*, and *rad52*Δ cells were used as a positive control for cells that were defective for GC. Unlike BIR, over-expression of *MPH1* did not affect the efficiency of gene conversion, as seen through robust growth on galactose plates (lower panel). *(D)* When no homologous MAT locus is available the HO cut must be repaired through NHEJ (upper panel). As expected, *yku70*Δ cells are defective for NHEJ and fail to grow on galactose plates, whereas overexpression of *MPH1* has no effect on NHEJ when compared to wild type cells harboring an empty vector (lower panel).

Based on its *in vitro* activity of D-loop displacement [18], we tested the notion that Mph1 may inhibit homology directed repair (HR) *in vivo.* We used a previously described reporter system [9]. Briefly, the reporter strain harbors an HO endonuclease recognition site on chromosome V that can be cleaved by a galactose-inducible HO endonuclease. The resulting DSB is flanked only on one side by a sequence (CA) that bears homology (A) to a template on chromosome XI (AN1). Due to this ‘one-sided’ homology the DSB can only be repaired through BIR and cells can only grow on galactose-containing plates after successful repair by BIR. The restoration of the *CAN1* gene leads to canavanine sensitivity, whereas loss of the *HPH* gene confers hygromycin sensitivity (Figure 1*A*). We show that *MPH1* overexpression specifically abolishes growth on galactose-containing plates and therefore conclude that Mph1 inhibits the repair of a double-strand break (DSB) via BIR (Figure 1*A, 1B*). Consistently, the repair frequency by BIR was slightly increased in cells carrying a *MPH1* deletion (Figure 1*B*, right panel). DSBs that are flanked on both sides by sequences that share homology to a template elsewhere in the genome get efficiently repaired by gene conversion (GC). If no homologous sequences are available, the DSB is repaired by NHEJ (non-homologous end joining). Strikingly, repair via GC (Figure 1*C*) and NHEJ [19] (Figure 1*D*) were not affected by Mph1 overexpression. As expected, GC and NHEJ were abolished upon deletion of *RAD52* and *YKU70,* respectively. Mph1 is a member of the DEAH family of ATP-dependent helicases [17]. The DEAH motif is required for the complete inhibition of BIR, as a DEAH-mutant that lacks ATPase activity *in vitro* [18] is a less potent inhibitor of BIR (Figure 1*B*).

**Figure 2:**
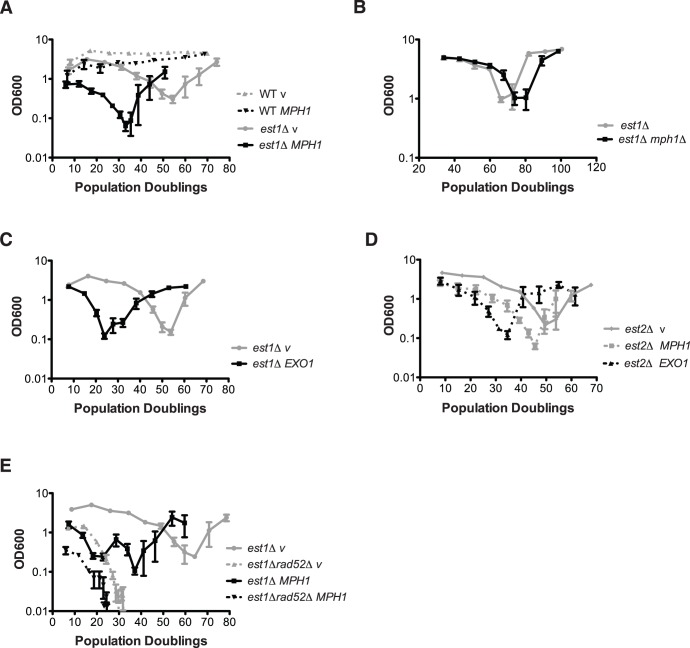
Mph1 causes pre-mature senescence. *(A)* An *est1*Δ*/EST1* heterozygous diploid was transformed with either empty vector or *MPH1* fused to a galactose inducible promoter on a 2µ plasmid. Cells were sporulated, microdisected and *MPH1* expression was turned on with 2% galactose. Liquid senescence assays were performed on the indicated genotypes at 30°C and diluted daily to an OD_600_ 0.01 (see materials and methods for detailed description). Mean and SEM are displayed. (*est1Δ v*: n = 5, *est1*Δ *MPH1*: n = 6, *WT v*: n = 4, *WT MPH1*: n = 4). *est1*Δ pGAL::*MPH1* cells lose viability faster than *est1*Δ *v* cells. The increased rate of senescence is not due to the toxicity of *MPH1* overexpression, as shown in wildtype (WT cells). *(B)* Deletion of *MPH1* delays senescence in *est1*Δ cells (*est1*Δ : n = 3, *est1*Δ *mph1*Δ: n = 7) cultured in YPD medium. *(C)* Overexpression of *EXO1* also leads to accelerated senescence in *est1*Δ cells (*est1*Δ *v*: n = 4, *est1*Δ *EXO1*: n = 6). *(D)* The *MPH1* and *EXO1* overexpression effects on senescence are also present in *est2*Δ cells (n = 4 for pGAL::*EXO1* and pGAL::*MPH1*, n = 3 for pGAL) *(E)* High-levels of Mph1 promote senescence even in the absence of homologous recombination (*rad52*Δ) and telomerase activity (*est1*Δ *v*: n = 2, *est1*Δ *rad52*Δ *v*: n = 4, *est1*Δ *MPH1*: n = 3, *est1*Δ *rad52*Δ *MPH1*: n = 3). For the latter assay *MPH1* was expressed from a galactose-inducible promoter on a centromeric plasmid.

As BIR has been implicated in the formation of survivors in the absence of telomerase activity, we monitored *est1Δ* mutant yeast cultures during senescence and recovery in the presence of *MPH1* overexpression. Est1 (ever shorter telomere 1) recruits the catalytic telomerase subunit Est2 to telomeres and is essential for telomerase mediated telomere elongation [21]. The optical density of the indicated yeast cultures (OD_600_) was plotted on the y-axis as an estimate of cell growth with respect to population doublings (PD) following a daily dilution/re-growth protocol (see materials and methods). Importantly, we confirmed that the OD_600_ measurement was an accurate representation of cell number through careful cell counting experiments (unpublished results). As expected *est1Δ* cells harboring an empty vector lose viability with increasing PDs due to telomere loss, however HR dependent survivors eventually take over the culture and restore nearly wild type growth rates (Figure 2*A* *, est1Δ* v). Surprisingly, *MPH1* overexpression did not inhibit survivor formation in an *est1Δ* mutant background, however it did accelerated the rate of senescence (Figure 2*A*). This effect was specific to cells lacking *EST1,* as there was no obvious loss of viability in wild type cells overexpressing Mph1 (Figure 1A). The continued expression of *MPH1* in survivors was confirmed by Western Blotting (Figure S1*A*). The typical appearance of multiple heterogenous telomeric bands on a Southern blot confirmed that type II survivors are being formed (Figure S1*B*), *i.e.* the telomeric tract has been amplified rather than the subtelomere as is the case for type I survivors. In contrast to overexpression of *MPH1*, deletion of *MPH1* slightly delayed the senescence in *est1Δ* cells (Figure 2*B*). We next asked whether another factor that inhibits BIR at a DSB, Exo1 [9], showed a similar tendency. Similar to *MPH1* overexpression, we observed faster senescence, without inhibition of survivor formation (Figure 2*C*) in an *est1Δ* culture when *EXO1* was overexpressed. Both *MPH1* and *EXO1* overexpression accelerated senescence without inhibition of survivor formation in *est2*Δ cells as well, ruling out that this effect was specific to *MPH1* overexpression in *est1*Δ associated senescence (Figure 2*D*). By analyzing the curves of each individual clone (Figure S1*C*) we realized, that although survivors were able to form, those that overexpressed *MPH1* or *EXO1* experienced greater instabilities in growth rate.

**Figure 3:**
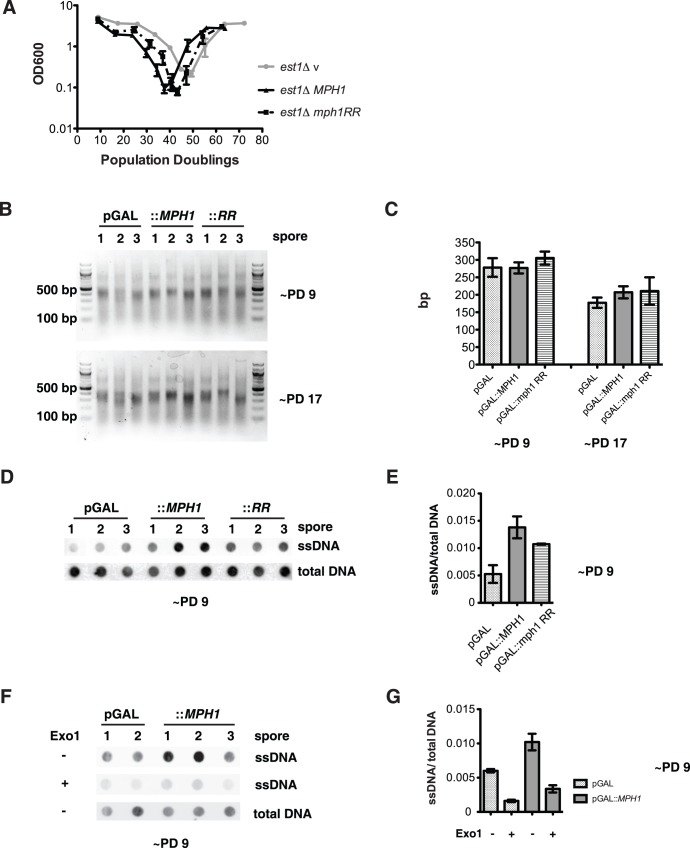
Mph1 does not affect telomere length, but increases the levels of ssDNA at the telomere. *(A)* The fast loss of viability phenotype in *est1*Δ depends on the DEAH domain, as the double arginine ‘*mph1 RR*’ mutant described in Figure 1*B* leads to a partial rescue of the early onset senescence. *(B)* Genomic DNA of the senescence assay described in *(A)* was extracted after about 9 and 17 Population doublings. The Y’ telomeres were amplified by PCR and run on an agarose gel. *(C)* Quantification of the PCR products does not reveal any difference in Y’ telomere length of *est1*Δ cells expressing either the empty vector, *MPH1* or the helicase mutant. *(D)* Genomic DNA was extracted under native conditions after about 9 population doublings. DNA of three independent cultures for vector control, *MPH1* and *mph1 RR* expressing cells was spotted on a nylon membrane. Telomeric ssDNA was revealed by annealing with a DIG-labeled telomeric C-rich oligonucleotide. DNA samples were denatured and submitted to dot blotting to control for equal loading. *(E)* The signal for ssDNA from *(D)* was quantified and normalized by the total amount of telomeric DNA. Overexpression of *MPH1* leads to an increased amount of telomeric ssDNA. This increase is partially suppressed upon overexpression of the *mph1 RR* mutant. *(F)* The ssDNA extracted under native conditions (Figure 3*D*) was digested for 2 hours with bacterial Exo1, which digests 3′-overhang DNA in the 3′ to 5′ direction. Samples were blotted on a nylon membrane and revealed by a DIG-labeled oligo-C-probe. Denatured DNA was used as a loading control. *(G)* Quantification of blotted ssDNA from *(C).*

Rad52 is required for most types of HR. The deletion of *RAD52* has been reported to compromise growth in an *est1Δ* background and leads to rapid senescence [22]. Surprisingly, *MPH1* overexpression was additive with the deletion of *RAD52* in the *est1Δ* background in terms of accelerating senescence (Figure 2*E*). This indicates that Mph1 promotes senescence through a mechanism that is independent of HR inhibition. Accelerated telomere shortening could potentially account for the faster senescence in *MPH1* overexpressing cells. We measured telomere length and found that bulk telomere length was not significantly changed in either *est1Δ* or wild type cells overexpressing *MPH1* compared to vector control cells, as determined by telomeric PCR amplifying telomere 1L (Figure S1*D*) and Y’ (Figure 3*B and C*). Similarly, the faster senescence in *est1Δ* cells overexpressing *EXO1* cannot be explained by loss of telomeric sequences, as there was no difference in length compared with control cells (Figure S1*E*). Nevertheless, we cannot exclude that *MPH1* overexpression leads to single short telomeres that could lead to premature senescence [23]. Together, the data presented in Figures 1 and 2 supports the notion that high cellular levels of Mph1 can prevent BIR at a DSB, but not at a telomere that is being maintained by HR. Moreover, overexpression of *MPH1* leads to a rapid senescence phenotype. However the inhibition of *RAD52* mediated HR does not account for the rapid senescence as *MPH1* overexpression increased the senescence rate even further in a *rad52*Δ genetic background. This indicates that another unidentified Mph1-mediated alteration at the telomere may be affecting senescence.

### Mph1 Overexpression Leads to the Accumulation of ssDNA at the Telomere

Resection of a double-stranded break leads to the accumulation of ssDNA that subsequently promotes HR. It has been previously reported that increased rates of senescence can be attributed to the accumulation of ssDNA at the telomere and the subsequent activation of a Mec1-dependent checkpoint [24]. We tested whether the accumulation of ssDNA at telomeres upon *MPH1* overexpression may be responsible for the rapid senescence and furthermore whether this depended on Mph1’s DEAH domain (Figure S2*A*).

Like the inhibition of BIR at a DSB, early onset senescence requires an intact DEAH domain, as overexpression of the *mph1 D209RE210R* mutant (Figure S2*B*) only partially accelerates senescence (Figure 3*A*). We extracted genomic DNA after 9 and 17 population doublings in galactose-containing medium and confirmed that in our *est1Δ* cultures bulk telomere length did not change due to overexpression of *MPH1* or *mph1 D209RE210R* (Figure 3*B and C*).

As aforementioned, we hypothesized that the high level of Mph1 could potentially lead to the accumulation of ssDNA at the telomere. Therefore we extracted genomic DNA from pre-senescent *est1*Δ cells from Figure 3*A*, and subjected it to a dot blot analysis under both non-denaturing and denaturing conditions (Figure 3*D*). Overexpression of *MPH1* led to a strong increase in the signal for telomeric G-rich DNA under native conditions in our *est1Δ* strain (Figure 3*D and E*). Denatured DNA served as a loading control. The ssDNA was quantified and normalized to the total amount of telomeric DNA spotted on the membrane (Figures 3*D and E*). Overexpression of the *mph1 D209RE210R* mutant showed an intermediate phenotype, indicting that the helicase domain is partially responsible for the accumulation of ssDNA at the telomere. In order to determine whether the ssDNA was due to an increased 3′ terminal overhang or the accumulation of internal DNA replication intermediates, we digested the native DNA with bacterial Exo1, which only removes terminal ssDNA in a 3′ to 5′ direction. The increase in ssDNA upon *MPH1* over-expression was strongly reduced upon digestion with Exo1 (Figures 3*F and G*) indicating that 3′ overhang accumulates upon *MPH1* over-expression. To further rule out that the ssDNA at the telomere stems from replication intermediates, we probed the native DNA with an oligo-G probe that hybridizes to the C-strand. We could not detect, any accumulation of ssDNA on the C-strand (Figure S2*C* and S2*D*). Together these data reveal that Mph1 leads to a telomeric alteration that becomes more accessible to nucleases in (uncapped) pre-senescent *est1*Δ cells.

### Overexpression of *MPH1* is Toxic in Mutants that are Defective in Telomere Capping

**Figure 4:**
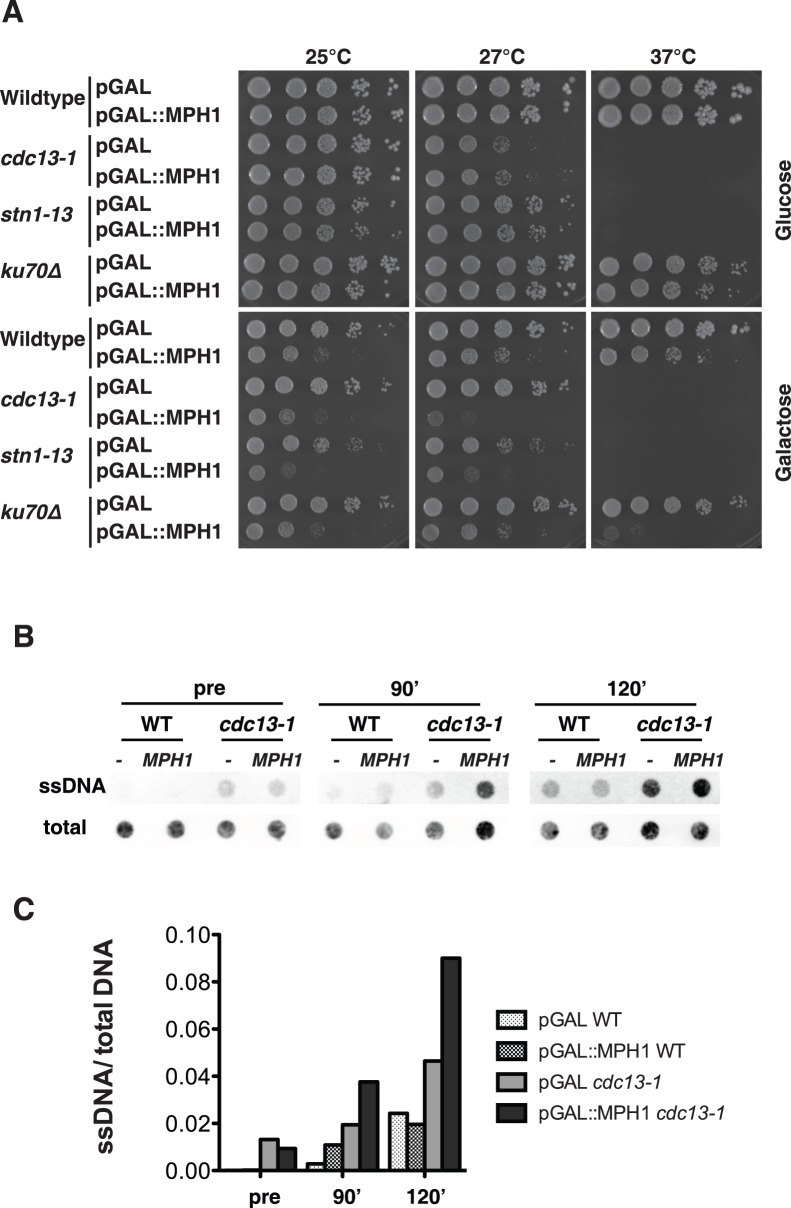
De-capped telomeres are sensitive to *MPH1* over-expression. *(A)* Galactose induced over-expression of *MPH1* causes lethality in *cdc13-1, stn1-13* and *ku70*Δ mutants at the respective semi-permissive temperature. *(B)* WT and *cdc13-1* cells either expressing the empty vector or *MPH1* fused to the galactose-inducible promoter were arrested with nocodazole for three hours and shifted to the semi-permissive temperature for the *cdc13-1* allele (27°C). Samples were taken prior to shift (‘pre’), 90 and 120 minutes after the temperature shift. DNA was extracted under non-denaturing conditions and spotted onto a nylon membrane. Telomeric ssDNA was revealed by annealing with a DIG-labeled oligonucleotide probe containing telomeric repeats. DNA was denatured and spotted on a nylon membrane and revealed as aforementioned for controlling the loading of total amounts of DNA. *(C)* The chemiluminescence signal of the blot in *(B)* was quantified and the ssDNA was normalized by the amount of total DNA.

Telomeres become uncapped with shortening of the telomeric tract. Telomere capping can also be promoted through two independent protein complexes: the CST (Cdc13, Stn1, Ten1) complex and the Ku70/Ku80 heterodimer. Upon inhibition of either the CST complex or the Ku70/Ku80 complex, the telomeric 5′ strand (C-rich) gets rapidly degraded in a manner that largely depends on Exo1. Since we have observed that overexpression of *MPH1* leads to uncapping in pre-senescent cells we predicted that mutants in the capping pathways may be particularly sensitive to *MPH1* overexpression. Indeed, overexpression of *MPH1* is toxic in *cdc13-1*, *stn1-13* and *ku70Δ* mutants at semi-permissive temperature (Figure 4*A*). In order to better understand the nature of *MPH1* toxicity in *cdc13-1* cells, we overexpressed *MPH1* in wild type cells and *cdc13-1* mutants from a galactose-inducible promoter in nocodazole for 3 hours at permissive temperature, to prevent differences in the samples arising from altered progression through the cell cycle. Cells were then shifted to the semi-permissive temperature for the *cdc13-1* allele (27°C) and samples were taken before the shift and both 90 and 120 minutes following the temperature shift. DNA was extracted under non-denaturing conditions and spotted onto a nylon membrane. The amount of ssDNA (G-strand) was detected by a C-rich telomeric probe and normalized by the total amount of telomeric DNA spotted (after denaturation). As expected [25] *cdc13-1* mutants accumulate ssDNA at non-permissive temperature (Figures 4*B and 4C*). Strikingly, we observed that overexpression of *MPH1* exacerbates the accumulation of ssDNA in the *cdc13-1* mutant background, whereas overexpression in wild type cells did not have an effect (Figure 4*B and 4C*). We tested several *mph1* point mutations for toxicity in *cdc13-1* (Figure S3). Surprisingly, mutants that are catalytically inactive *in vitro* like E210Q or D209NE210Q still decreased the viability of a *cdc13-1* and a *cdc13-1 mph1*Δ strain (Figure S3*B* and S3*C*). Inversion of the electric charge in *mph1 D209RE210R* led to a decrease in toxicity. Wild type as well as *mph1* mutant proteins were expressed at similar levels, as assessed by Western Blotting (Figure S3*D*).

### Mph1 Inhibits NHEJ-dependent Fusions at the Telomere

**Figure 5:**
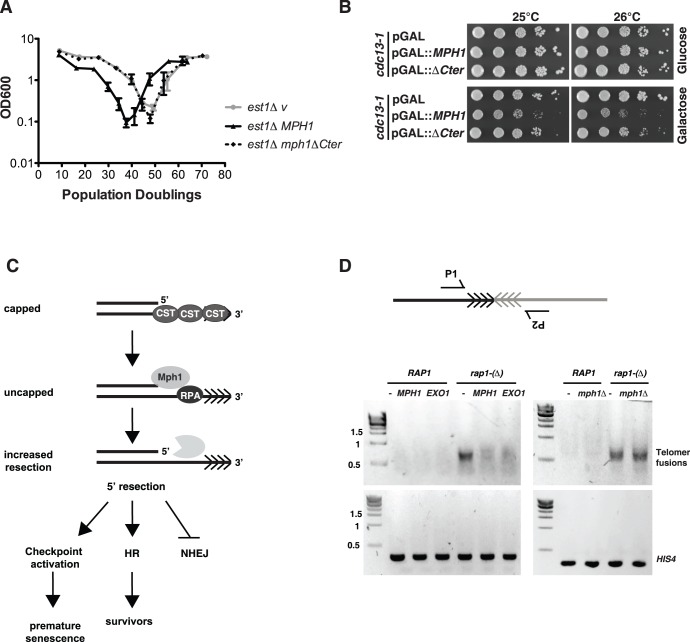
Model: Mph1 promotes HR by enhancing resection and thereby inhibits NHEJ. *(A)* Mph1 interacts with the ssDNA binding protein RPA via the C-terminal of Mph1. Deletion of the C-terminal part of Mph1 completely abolishes the acceleration of senescence in *est1*Δ cultures. *(B)* Overexpression of *mph1ΔCter* is less toxic than overexpression of the wildtype *MPH1* in a *cdc13-1* temperature-sensitive strain. Strains were grown at semi-permissive temperature under inducing (galactose) and non-inducing conditions. *(C)* Whether a cell undergoes repair by HR or NHEJ is decided at the level of resection, as a ssDNA overhang is required for HR, but not for NHEJ. We propose that Mph1 leads to increased telomeric resection at a dysfunctional telomere and thereby promotes recombination, *i.e.* faster rate of survivor formation. This leads to the prediction that *MPH1* overexpression should limit chromosome end-to-end fusion, which arise via NHEJ. *(D)* Strains of the indicated phenotypes were allowed to reach stationary phase by growing them for 5 days in liquid culture. A strain containing the Rap1 degron allele (*rap1-(Δ* served as a positive control for high levels of chromosome end to end fusions. In this strain Rap1 function is impaired in stationary phase at 30°C [20]. Overexpression of *MPH1* and *EXO1* from a constitutive GPD-promoter containing plasmid decreased the amount of chromosome end-to-end fusions fusions between Y’ telomeres in a decapping *rap1-(Δ* mutant. Deletion of *MPH1* had no effect on the occurrence of telomeric fusions. Fusions were detected via telomere PCR and agarose gel electrophoresis. PCR of the *HIS4* gene served as loading control.

The C-terminus of Mph1 has been shown to interact with RPA [16]. RPA’s primary function is to stabilize ssDNA. As overexpression of *MPH1* leads to increased levels of ssDNA at telomeres and hence faster senescence, we wondered whether the interaction between Mph1 and RPA was crucial to promote senescence. To test this hypothesis, we overexpressed *MPH1* and an *mph1* mutant lacking the C-terminal residues crucial for interaction with RPA [16] (*mph1ΔCter*, Figure S4*A* and S4*B*). Indeed overexpression of *mph1ΔCter* in an *est1Δ* background did not accelerate senescence in comparison with high levels of full-length *MPH1* (Figure 5*A*). Consistently, the overxpression of the *mph1*Δ*Cter* is not as toxic in *cdc13-1* cells as compared to overexpression of the full length Mph1 protein (Figure 5*B* and Figure S4*C* for Western Blot). Taken together, the interaction between Mph1 and the ssDNA binding protein RPA is crucial to inhibit the growth of cells with impaired telomere integrity.

We hypothesized that increased resection at the telomere could potentially promote HR and concomitantly inhibit NHEJ (Figure 5*C*) since NHEJ requires a blunt end for ligation. NHEJ can lead to deleterious fusions between telomeres and has to be suppressed at chromosome ends in order to conserve genome integrity. We used a previously described PCR-based approach to test for NHEJ-dependent fusions between [20]. Only if two telomeres are fused, can they be amplified by PCR using two forward primers (P_1_ and P_2_) specific for different chromosome ends (Figure 5*D* upper panel). A sensitized *rap1-(Δ)* background was used, as this strains shows a high incidence of inter-telomeric fusions [20] (Figure 5*D*, lower panel). Indeed, overexpression of *MPH1* drastically reduced the amount of chromosome end-to-end fusions in the *rap1-(Δ)* strain (Figure 5*D* and Figure S4*D*). Overexpression of the exonuclease, *EXO1,* phenocopied our observations with *MPH1* overexpression whereas the deletion of *MPH1* did not lead to more fusions (Figure 5*D*, right panel). PCR of the *HIS4* gene was used to verify equal input of genomic DNA for the PCR reaction. Thus high levels of Mph1 and Exo1 inhibit telomere fusions, but on the other hand lead to increased levels of ssDNA and growth arrest in cells bearing dysfunctional telomeres.

## Discussion

Mph1 seems to play the role of a double-edge sword at the telomere: while overexpressed *MPH1* can inhibit deleterious fusions between chromosome ends, it can also cause growth inhibition and rapid senescence in cells defective for telomere capping. The underlying mechanism seems to be the regulation of ssDNA levels. High ssDNA levels promote HR due to the accumulation of invasive Rad51-coated molecules whereas NHEJ-dependent fusions are inhibited by ssDNA due to the requirement of a blunt end. We hypothesize that the levels of Mph1 have to be kept in check at the telomere in order to establish the correct balance between senescence, HR and NHEJ inhibition, ensuring that the above mentioned processes are either inhibited or promoted at the right time according to the functional state of the telomere. It has been previously shown that the levels of the human homolog of Mph1, FANCM, are controlled via ubiquitin-dependent degradation after DNA damage [26] and in mitosis [27]. Therefore, it will be important to investigate, whether Mph1 levels are also regulated via ubiquitination at the telomere.

Interestingly, FANC proteins have recently been shown to inhibit NHEJ at DSBs and drive repair towards HR, much like what we have seen with Mph1 at the telomere. Indeed FANCD2 was proposed to compete with the NHEJ-factor Ku70 for access to DSBs in both chicken and human cells [28]. Furthermore, the FANCD2 homolog in *C.elegans* has been reported to prevent erroneous repair of DSBs by NHEJ during meiosis, again by promoting HR [29]. Thus, FANC proteins seem to be instrumental in regulating the choice of repair pathway at a DSB. In this respect it may not be surprising that the FANC proteins also have an important role at chromosome ends, as telomeres structurally resemble DSBs in many respects [30]. Whereas, the inhibition of NHEJ at telomeres is essential in order to prevent chromosome fusions, a source of genomic instability, NHEJ is often the choice of repair at a DSB. Our results suggest that Mph1-mediated repair decisions at telomeres are differently regulated from repair at a DSB. While *MPH1* overexpression potently inhibits NHEJ at the telomere (Figure 5*D*), it does not affect NHEJ-mediated repair at an internal DSB (Figure 1*D*). Along the same lines, *MPH1* can completely abolish BIR at a DSB, however allows recombination (survivor formation) to occur at the telomere. Consistent with the notion that *MPH1* does inhibit HR at telomeres, we found that *MPH1* overexpression in *rad52Δest1Δ* cells exacerbated the early onset of senescence (Figure 2*E*). The abnormally high levels of ssDNA in *est1Δ* mutant cultures overexpressing *MPH1* (Figure 3*D and 3E*) are likely accountable for the early senescent phenotype. With *MPH1* overexpression we found no evidence of excessive bulk telomere shortening, although we cannot rule out that single short telomeres may arise more frequently.

How could the action of Mph1 lead to increased levels of telomeric ssDNA? In mammalian cells telomeres have been shown to form a lasso-like structure (T-loop) [31], where 3′overhang invades the telomeric dsDNA, resulting in the formation of a Dissociation (D)-loop. Yeast telomeres have also been shown to form a fold-back structure [32], [33], [34], [35] although the characteristics of these loop structures are less well described than those of mammalian T-loops. Mph1 has been shown to undo D-loops *in vitro* [18], via its helicase activity. Although the function of T-loops remains enigmatic, they have been proposed to play a protective role, by sequestering the telomeric overhang and thereby preventing checkpoint activation and unscheduled recombination. Interestingly, Mph1 has the same *in vitro* activities as RTEL1 (Regulator of telomere elongation) and RTEL1 has been recently shown to open T-loops *in vitro* in an ATP-ase dependent manner [36]. FANCM is another candidate for a T-loop regulator as it can also disassemble D-loops *in vitro* [37]. It will be interesting to determine whether Mph1, FANCM and RTEL1 have the ability to affect T-loops structure *in vivo*. The disassembly of T-loops could explain how exonucleases gain increased access to telomeres and thereby increase the levels of ssDNA when *MPH1* is overexpressed. We propose that Mph1 may have two mechanisms of function *in vivo* one depending on its helicase activity, probably by undoing a D-loop intermediate and the other one being a scaffolding function independent of its ATPase/helicase activity possibly by recruiting and or stimulating nucleases. Interestingly, it has been published that Mph1 stimulates the endonuclease activities of both Fen1 and Dna2 *in vitro* [38] and that this stimulation in independent of the ATPase/helicase activity of Mph1. This would be in agreement with our observation that over-expression of helicase-dead Mph1 still leads to slightly elevated levels of ssDNA at the telomere in *est1Δ* cells (Figure 3*D and E*). Another, less likely, possibility would be that the helicase dead mutants are still active to an extent *in vivo,* despite their full loss of activity *in vitro.* It is noteworthy that the proposed human homolog of Mph1, FANCM, also plays a dual role: the role in checkpoint activation and replication fork progression depends on its ATPase activity [31], [39], whereas the recruitment of the Fanconi anemia core complex to chromatin (its scaffolding function) occurs independent of its ATPase domain [40].

The RTEL1 locus is amplified in many human tumors and upregulation of RTEL1 led to liver malignancies in mice [41]. Similarly, FANC proteins including FANCM were found to be transcriptionally upregulated in melanomas [12]. Furthermore, the FANC pathway was implicated in resistance to chemotherapeutic agents [13], [14]. Therefore, we think it is crucial to not only understand the mechanism of action of these helicases, but also how to keep them in check.

## Supporting Information

Figure S1 *(A)* Western Blot showing the expression of *MPH1*-HA in two independent cultures of *est1Δ* survivors. Actin served as loading control. *(B*) DNA of survivors was extracted, digested by XhoI and analyzed by Southern blotting using a G-rich telomeric probe. The appearance of the smeared multiple bands in the vector-control (n = 3) and the *MPH1*-overexpressing cells (n = 3) shows that an amplification of telomeric repeats has occurred. This is typical for typeII survivors. *(C)* Individual curves of each culture expressing either empty vector of *MPH1* or *EXO1* from a galactose-inducible promoter. *(D)* Quantification of telomere length PCRs for telomere 1L corresponding to the senescence assay presented in Figure 2*A* (n = 3 for *est1Δ v* and *MPH1*, n = 2 for WT *v* and *MPH1*). *(E)* Quantification of Y’ telomere length by PCR for the senescence assay in *est1Δ* cells overexpressing *EXO1* (n = 3 each). Overexpression of *EXO1* does not change telomere length in an *est1*Δ background. *(F)* Quantification of Y’ telomere length for the senescence curves of the *est1Δ rad52Δ* survivor assays (n = 2 each). As published previously, the telomere length in *est1*Δ *rad52*Δ is slightly longer than in *est1*Δ cells. All telomere lengths were calculated by subtracting the amount of sub-telomeric DNA from the total length of the PCR product, giving the length of the TG_1-3_ tract exclusively. All graphs display mean and SEM.
(EPS)

Figure S2 *(A)* Cartoon depicting the Mph1 protein. The D209RE210R mutations alter the DEAH motif. *(B)* Western Blotting confirming the expression of *MPH1*-HA and *mph1D209RE210R*-HA in *est1Δ*cells after about 25 population doublings. Proteins of two different clones (Figure 2*D*) were extracted. Ponceau staining serves as loading control. *(C)* Genomic DNA of the senescence curve presented in Figure 3*A* was blotted on a nylon membrane and hybridized with a DIG- labeled oligo-G-probe under native conditions (top panel) or denaturing conditions (bottom panel). Conversely to what has been observed in Figure 3*D and E* for the G- rich overhang, there was no accumulation of ssDNA of the C-rich strand detected. *(D)* Quantification of ssDNA on the C-strand *(D)* and normalization by the amount of total denatured DNA.
(EPS)

Figure S3 *(A)* Cartoon depicting the DEAH and Helicase of Mph1 and the respective positions of the point mutations used in this study. K113Q lies within the ATPase domain, D209 and E210 are residues of the DEAH helicase motif. *(B)* Overexpression of wild-type and *mph1* point mutations in *cdc13-1* under semi-permissive conditions. Only the *mph1 D209RE210R* shows a reduced toxicity in the *cdc13-1* background. *(C)* Overexpression of *mph1 D209RE210R* is the least toxic in a *cdc13-1 mph1Δ* background at semipermissive temperature. *(D)* Western Blot showing that HA-tagged wildtype and mutant Mph1 are expressed at similar levels in a *cdc13-1 mph1Δ* background. Ponceau staining serves as loading control.
(EPS)

Figure S4 *(A)* Cartoon depicting the Mph1 protein lacking the C-terminal amino acids responsible for binding to RPA. *(B)* Western blotting confirming the expression of the *mph1-ΔCter*-HA mutant protein in an *est1Δ* background after about 25 population doublings. Ponceau staining serves as loading control. *(C)* The *mph1-ΔCter*-HA mutant protein is expressed in a *cdc13-1* mutant strain. *(D)* Serial dilutions of strains grown for 5 days to stationary phase were spotted onto synthetic medium containing either glucose or galactose. *MPH1* and *EXO1* were constitutively expressed from a GPD-promoter containing plasmid. Galactose-induced inactivation of the centromere on chromosome 6 allows cells containing a telomere end-to-end fusion to grow stably, rather than suffering from genomic instability [20].
(EPS)
